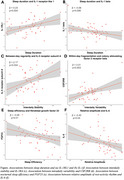# Sleep and 24‐hour rhythm associations with plasma markers of inflammation in aging

**DOI:** 10.1002/alz70856_105983

**Published:** 2026-01-10

**Authors:** Skylar E Weiss

**Affiliations:** ^1^ Stanford University, Stanford, CA, USA

## Abstract

**Background:**

Disrupted sleep and 24‐hour rhythms contribute to age‐related inflammation, a key pathway influencing vulnerability to chronic disease and mortality in older adults. Previous studies have explored associations between lifestyle measures and inflammation, but additional research is needed to characterize associations between sleep‐wake rhythms and inflammatory cytokines in the context of healthy brain aging.

**Method:**

54 cognitively normal older adults (77.2  ±  6.6 years, 57% female) completed blood draws and 14 days of at‐home actigraphy (wrist‐worn accelerometry). Plasma samples were analyzed using the Alamar NULISASeq Inflammation panel. Actigraphy analyses focused on nocturnal sleep efficiency, sleep duration, 24‐hour relative amplitude, interdaily stability, and intradaily variability. Sixty cytokines and other proteins were selected based on their roles in regulating inflammation. Associations between actigraphy measures and inflammatory markers were tested using linear regression with age, sex, and time between collection (2.0  ±  2.8 years) included as covariates. Multiple comparisons corrections were not used. NULISA *p*‐tau217 was added as a covariate in sensitivity analyses.

**Result:**

Worse sleep and 24‐hour rhythms were generally associated with an elevated inflammatory cytokine profile (Figure). Shorter sleep duration was associated with lower levels of IL‐1 receptor‐like 1 and higher levels of IL‐1β. Day‐to‐day regularity of 24‐hour rhythms (interdaily stability) was positively associated with IL‐5 receptor subunit A, and within‐day fragmentation (intradaily variability) was positively associated with colony stimulating factor 2 receptor beta. Worse sleep efficiency was associated with higher fibroblast growth factor 23. Higher relative amplitude of 24‐hour rhythm was associated with lower IL‐6. When *p*‐tau217 was added as a covariate, associations remained significant for IL‐1β, IL‐6, IL‐5RA, FGF23, and CSF2RB, but not for IL‐1RL1, suggesting that these associations were independent of Alzheimer's disease pathology.

**Conclusion:**

These preliminary findings support the hypothesis that disrupted sleep and 24‐hour rhythms are associated with higher pro‐inflammatory and lower anti‐inflammatory cytokines among healthy older adults. Future work is needed to examine how associations between sleep and inflammation impact cognitive aging trajectories.